# Supplementary material for: Are carbon emissions trading and green financial instruments synergistic? -Comprehensive quantitative research based on content analysis
Source: PLoS One. 2024 Mar 7;19(3):e0298601. doi: 10.1371/journal.pone.0298601 (PMC10919601; doi:10.1371/journal.pone.0298601)
Supplement: S1 Data — (ZIP) [file pone.0298601.s001.zip › Data/地级市Z_F工作报告.zip!/║■─╧/┬a╡╫╩╨/└┤╫╘╣1⁄2╓┌║┼í╛┬φ┐╦╩2╛▌═°í┐_2010.docx]

各位代表：现在，我代表市人民政府向大会作工作报告，请予审议，并请各位政协委员和其他列席人员提出意见。

一、2009年政府工作回顾

　　2009年是新世纪以来我市经济发展最为困难的一年，也是我们经受严峻考验并取得重大成就的一年。 　　面对国际金融危机的严重冲击，市政府在市委的坚强领导下，在市人大、市政协的监督支持下，深入学习实践科学发展观，认真贯彻落实中央和省里的一系列决策部署，牢牢把握“保增长、扩内需、调结构、促就业、强基础”的总体要求，突出“工业主导、基础先行、项目带动、协调发展”的战略重点，团结一致，奋力攻坚，较好地完成了年度目标任务，努力开创了我市“科学发展，加速赶超”的新局面。 　　全年实现地区生产总值570亿元，增长12.6%（预计数，下同）；完成财政总收入45.51亿元，增长13.6%，其中一般预算收入25.53亿元，增长14.3%；城镇居民人均可支配收入13422元，农民人均纯收入3280元，分别增长8.9%和9.3%；人口自然增长率7‰；城镇登记失业率4.5%；亿元GDP生产安全事故死亡率0.35；万元GDP综合能耗降低4.5%，化学需氧量削减4.97%，二氧化硫、砷排放量提前完成“十一五”规划目标。 　　（一）工业经济较快增长 　　受国际金融危机影响，去年上半年我市规模工业增加值增速由年初的9%一度下滑至5月份的3%，全市生产总值增速同步下滑至7.2%。在严峻的形势面前，市委、市政府果断决策，科学应对，力促规模工业止跌回升，全年增速达到17.8%，总计完成工业增加值269亿元，增长16.1%。 　　工业企业加快发展。及时出台促进企业平稳较快发展若干意见，集中开展“工业企业服务年”活动，由市级领导牵头，上千名干部深入企业开展服务，帮扶企业促技改、调结构、拓市场、渡难关。完成工业投资169亿元，增长30%，其中技改投资140.5亿元，增长20.7%。原煤、发电量、钢材、焦炭、锑品、水泥等主要产品产量分别增长10%、34%、25%、36%、54%、46%。十大产业集群专项规划编制完成，实现增加值190.5亿元，增长19.2%。规模工业企业总户数达到601户，新增92户。 　　园区工业不断壮大。研究出台支持产业园区发展的政策措施，完善园区绩效考核办法，明确园区高技术产业发展定位，实现园区工业增加值63.4亿元，增长16%。娄底经济开发区获批“国家高技术产业基地娄底薄板材料深加工产业园”，各县市区产业园也竞相发展。 　　创新能力明显提升。扎实开展“科技创新年”活动，推进产学研结合，市内75家企业与国内35所高校和科研院所成功对接，在全省率先组建了湖南特种陶瓷、煤矿机电、农业机械、特色生猪等四大产业技术创新战略联盟。闪星锑业技术中心进入国家级技术中心行列。新增高新技术企业16家、湖南名牌产品8项，争取省级以上科技项目51项。市农科所选育的杂交水稻新品种“陆两优267”通过省级鉴定，在全省大面积推广应用。我市顺利通过国家知识产权试点城市创建考核验收，获批“国家高技术产业基地”和“湖南省综合性高技术产业基地”，冷水江成功创建全国科技进步先进市。 　　（二）投资拉动强劲有力 　　抢抓国家实施扩内需一揽子计划的有利机遇，大力推进“项目建设年”活动。全年施工项目1276个，增长48.2%，其中新开工项目1010个，增长48.1%。完成全社会固定资产投资311亿元，增长54.2%，是我市历史上投资规模最大、增速最快的年份之一。 　　工业建设呈现新亮点。涟钢2250热轧板生产线、金电二期、华润电力一期、双峰海螺二期、湖南海螺、安石湖南煤化新能源等30个重大项目竣工投产，72个重大项目开工建设，尤其是总投资115亿元的华菱安赛乐米塔尔汽车板电工钢项目成功落户娄底经济开发区并开工建设，将为我市发展增添强大后劲。 　　交通建设取得新突破。完成投资34.32亿元。成功争取沪昆铁路客运专线通过我市，并规划在娄底城区和新化设站，娄邵铁路扩能改造顺利开工。娄新、新溆、安邵三条高速公路建设全面推进，打响了干线公路改造三年大会战，加快推进G207、S210、S312、S209、S217、S225、X024等七条干线公路改造，娄底汽车南站投入使用，农村通乡通畅工程完成1231公里，其中改造县乡公路140公里，新建乡镇到村水泥路1091公里。全面实施成品油价格和税费改革，交通管理体制和运行机制初步理顺。 　　城市建设迈出新步伐。完成投资18.6亿元。市城市建设投资集团有限公司正式运营，成为城市投资的主载体、城市融资的主平台和城市建设的主力军。中心城区吉星路等主次干道完成提质改造，孙水公园建成开放，市体育中心和涟水河南岸防洪堤主体工程竣工，城区绿地率达到34.25%。县市城区与小城镇加快建设。房地产业健康发展，全年商品房施工面积、竣工面积分别增长21.2%和10.7%。成功争取水府示范片区纳入长株潭城市群昭山示范区范畴，与长株潭对接融入迈出了坚实的一步。 　　要素保障得到新加强。加快对接国家、省产业调整和振兴规划，40个重大项目进入省重点项目规划，17家企业列入省重点产业骨干企业名录，争取中央、省各类建设资金26.1亿元，比上年增加11.1亿元。积极搭建对外合作交流与招商引资平台，成功举办娄底深圳投资洽谈会、广州科学城投资推介会、第三届“青春娄底·湘中之光”经贸洽谈会，组团参加“中博会”、“港澳洽谈周”、“珠洽会”、“湘商大会”，实现内联引资80.5亿元，实际利用外资1.34亿美元，是我市历史上招商引资力度最大、措施最硬、效果最好的一年。大力实施金融机构扶持中小企业600家工程，组建了2个资本金过亿元的担保公司，成立了2家小额贷款公司，冷水江成功创建省级金融安全区。全市金融机构各项贷款余额比年初净增97.2亿元，增量居全省第四位，增速居全省第一位。重点项目用地得到有效保障，连续十年实现耕地总量占补平衡，维护矿产资源管理秩序工作荣获全国先进。 　　（三）农业基础更加巩固 　　落实强农惠农政策，农业经济稳步增长。全年完成农业增加值92亿元，增长5%，粮食播种面积393.5万亩，总产162万吨，分别增长4.29%、3.19%。出栏生猪499.7万头，增长3.4%。发放粮食直补、良种补贴、化肥补贴、农机购机补贴、种粮大户补贴等补贴资金3.22亿元。全市“三农”支出完成8.32亿元，增长29.78%。种养大户发展到5.1万户，农民专业合作组织达到372个，会员7.2万户，带动农户19.26万户。农产品加工企业达到888家，完成产值47.8亿元，增长20.6%。双峰、新化分别获全国粮食生产标兵县和全省粮食生产先进县称号，新化、涟源列入国家油茶基地建设重点县（市）。 　　推进新农村建设，农业基础不断夯实。重点抓了78个省级新农村示范村建设，逐步形成了特色产业型、生态养殖型、劳务经济型等8种建设模式，起到了良好的示范带动作用。完成水利建设投资3.9亿元，完成各类水利工程5.1万处，解决24.4万农村人口饮水安全问题，水利建设工作获全国先进，第六次获得全省水利建设竞赛“芙蓉杯”奖。农业机械化率达到27.1%，农机产值达到10.7亿元，已有27个产品纳入国家和省支持推广的农机产品目录。扶贫建设、革命老区、库区开发、移民后扶、农民负担监管、“三冬”生产等工作扎实开展。及时有效抗御山洪灾害，夺取了双峰“7·2”抗洪救灾胜利。森林防火、农林病虫害防治、气象水文信息和技术服务、农资供应与价格调控、农产品质量监管等工作进一步加强，动物疫病防控工作进入全省前列。农村道路、通信等设施不断完善，农网改造完成投资1.22亿元。加强基本农田保护，新化被评为全国基本农田保护、农业综合执法先进县。推进土地集约经营，已流转耕地面积31.5万亩，占承包面积的15.4%。狠抓农村能源建设、乡村清洁工程和农村面源污染的整治，改善了农村生态环境。集体林权制度改革稳步推进，林地现场核实率、林权发证率、山林纠纷调处率分别达到92.1%、36.1%和88.4%，均超过省定年度目标。完成造林11.6万亩，新增油茶林2.2万亩，森林覆盖率达到47.5%。 　　（四）促进消费成效显著 　　全年完成第三产业增加值187亿元，增长11.2%，实现社会消费品零售总额185.6亿元，增长18.7%。我市成为全国首批商务综合执法试点城市，“万村千乡市场工程”、“双百市场工程”持续推进，“家电下乡”、“汽车摩托车下乡”活动效果明显，发放财政补贴9800万元，带动直接消费15亿元。成功举办湖南省首届农业机械、矿山机械、电子陶瓷产品博览会，加工贸易实现零的突破，进出口总额居全省第四位。消费者权益得到有效保护。居民消费价格总水平上涨0.7%。旅游业快速发展，旅游接待总人数达到544万人次，增长34.5%，实现旅游总收入27.6亿元，增长36.4%。启动了大梅山文化旅游区域合作，开展了“亿元优惠迎国庆·万人免费游娄底”大型促销活动，成功举办第二届曾国藩国际文化旅游节，波月洞成功创建AAA景区，梅山龙宫、曾国藩故居创AAAA景区通过国家专家组评审，填补了湘中AAAA景区的空白。湄江成功申报国家级地质公园，紫鹊界被评为国家水利风景区，我市创建中国优秀旅游城市通过国家验收。 　　（五）民生保障力度加大 　　为民办实事任务圆满完成。实施33个为民办实事项目，完成投资19亿元。落实积极就业政策，开展就业培训10.7万人，新增城镇就业和安置再就业5.1万人，稳定农村劳动力转移就业107.4万人。养老、失业、医疗、工伤和生育保险覆盖面不断扩大，全市社会保障支出18.71亿元，增长48.88%。新型农村养老保险在娄星区启动试点。新型农村合作医疗基金运行效益和参合农民受益水平提高，参合农民280.8万，参合率达到85.37%。残疾人事业加快发展。新增廉租住房7167套，新建经济适用房1298套。加强财政、政府投资项目和专项资金审计，确保了资金使用效益和惠民政策落实。 　　节能减排扎实推进。强化环境监管，严格控制高耗能、高污染项目，坚决淘汰落后产能，关闭“三高一低”企业和项目32个。市第二污水处理厂主体工程基本完成，4个县市污水处理厂全部建成投入运行。 　　社会大局和谐稳定。强化安全生产责任落实，加大隐患排查治理力度，各类安全事故持续下降。争取中央投资2374万元、银行贷款3亿元，实施国家煤矿安全改造项目12个，煤矿安全生产消灭重大及以上事故，创历史最好水平。推进“百件信访老案”办理，集中解决了大批遗留问题。调整警务体制，加强防控建设，刑事发案率进一步下降，社会治安综合治理取得明显进步，信访和维稳工作获得全省先进。 　　各项社会事业协调发展。教育事业加快发展，基础教育进一步巩固，职业教育、成人教育、高等教育协调发展。城区学校入学新生班额过大问题得到有效控制，高中招生制度改革初见成效，娄底技师学院筹建工作扎实推进。我市顺利通过国家二类城市语言文字工作评估。加快公共卫生服务体系建设，全面推进医药卫生体制改革，启动实施六项公共卫生服务均等化项目和娄星区、冷水江基本药物制度试点工作，社区卫生服务机构实现全覆盖，医疗卫生服务水平全面提升。甲型H1N1流感得到有效防控。药监工作获全国医药卫生系统先进。慈善、老龄和红十字会工作卓有成效。人口和计划生育工作连续三年获全省先进，我市被确定为全国首批人口文化新传播基地。大力发展竞技体育和全民健身运动，人民群众身体素质不断增强。组织“中国著名作家看娄底”大型采风创作活动，大型现代花鼓戏《花落花开》获得田汉戏剧奖并被列入全省农村党员远程教育教材，电影《马东的假期》荣获全国中小学生优秀影片奖，新闻出版业加快发展，乡镇综合文化站、广播电视村村通和数字电视工程建设扎实推进，市广电技术大楼投入使用。组织了建国60周年和撤地设市10周年系列庆祝活动，彰显了“青春娄底”形象，激发了全市人民的自豪感，进一步凝聚了全市人民科学发展、加速赶超的合力。 　　全面推进国企改革改制扫尾攻坚，市属106家企业已有102家完成改制，省属下划企业湖南省锻造厂改制完成。 　　军队和国防后备力量建设稳步推进，公安、消防、武警队伍建设不断加强，在全市经济社会发展特别是抗灾救灾和维护社会稳定中作出了重要贡献。统计服务质量明显提高，侨务工作获全国侨务系统先进，人防工作扎实有效，民族、宗教、地震、对台、地方志、档案、保密和工会、共青团、妇联等各项事业协调发展。 　　（六）自身建设不断加强 　　主动接受人大法律监督和政协民主监督，认真办理市三届人大一次会议议案2件、人大代表建议209件，政协建议2件、政协委员提案196件。支持各民主党派、工商联、无党派人士参政议政。贯彻落实《湖南省行政程序规定》，出台《娄底市人民政府重大行政决策程序规则》，聘请了68名专家，开展重大行政决策事项咨询论证工作，加强行政复议和规范性文件管理，有力推进了依法、科学、民主决策。推进政府机构改革和人事制度改革，全面实施政府绩效评估，切实加强行政监察和政务督查，严格实行行政问责，大力实施投资项目全程代理服务和建设项目行政审批收费“一票制”，机关效能建设逐步加强。集中整治“三强”（强装强卸、强揽工程、强行阻工）行为，着力优化施工环境。深入开展学习实践科学发展观活动，干部作风进一步改进。加强财税征管，促进财政增收，开展厉行节约工作，财政保障能力增强。严格执行党风廉政建设责任制，坚持不懈加强反腐倡廉建设。切实加强基层民主政治建设，全市第七次社区居委会换届选举圆满完成。 　　各位代表，回顾过去一年的工作，我们不仅取得了各项建设的重要成果，也积累了在复杂环境中推动经济社会又好又快发展的重要经验。一是必须坚定不移地实施科学发展、加速赶超总体战略，以切实可行的思路引领发展，全力以赴促进发展。二是必须立足实际，选准路径，在比较中学习，在学习中提升，不断增强推动科学发展的能力。三是必须解放思想，开放视野，胸怀全局，科学研判，不断增强工作的预见性、主动性和针对性。四是必须不畏艰难，敢于担当，大力弘扬“坚韧、勤奋、尚德、自强”的娄底精神，始终保持奋发有为的精神状态。五是必须高度重视抓基层、打基础，夯实发展平台，优化发展环境，增强发展后劲。 　　经验弥足珍贵，成绩来之不易。这是市委总揽全局、科学决策的结果，是市人大、市政协有力监督和社会各界全力支持的结果，是各级各部门扎实工作和全市人民努力奋斗的结果。在此，我谨代表市人民政府，向全市人民，向各位代表和委员，向各民主党派、工商联、无党派人士、各人民团体和社会各界人士，向驻娄人民解放军、武警和消防官兵、政法干警，向各位离退休老领导、老同志，以及所有关心、支持我市建设和发展的朋友们，表示衷心的感谢并致以崇高的敬意！ 　　在肯定成绩的同时，我们也清醒地看到，我市发展中也存在很多困难和问题，主要是：经济实力还不强，区域竞争压力大；产业结构还不合理，转变发展方式任务艰巨；节能减排要求高，生态建设和环境保护任重道远；经济发展环境还不优，转变政府职能、提高行政效能任务很重；老百姓关心的一些热点、难点问题亟需解决，改善民生力度还需加大。对这些问题，我们必须增强责任感和紧迫感，在今后的工作中切实加以解决。

二、2010年政府工作目标与主要任务

　　2010年，是实施“十一五”规划的最后一年，也是形势最复杂的一年。要看到，国际国内经济发展形势逐步好转，特别是中央继续实施积极的财政政策和适度宽松的货币政策，有利于我市争项目、扩投资；中央把加快经济发展方式转变作为今年经济工作的重点，有利于我市调结构、促转变；国家进一步促进中部地区加快崛起，长株潭城市群“两型”社会建设深入推进，为我市发展提供了更多的机遇，拓展了更广的空间；我市经过国际金融危机洗礼，积累了经验，抵御风险的能力进一步提高。同时，也要看到，国际金融危机影响仍然存在，宏观环境纷繁复杂，各种不确定因素仍然较多；省内兄弟市州竞相发展，我市与长株潭等先进市州的差距拉大，赶超的压力增加。对此，我们既要增强忧患意识，更要坚定发展信心，下更大决心，花更大力气，采取更加有效的措施，确保完成各项目标任务。 　　今年政府工作的总体要求是：以党的十七大、十七届三中和四中全会以及中央、省委经济工作会议精神为指针，深入贯彻落实科学发展观，按照市委三届六次全会和市委经济工作会议的总体部署，深入实施“科学发展，加速赶超”战略，坚持工业主导、基础先行、项目带动、协调发展，以学习、对接、融入、服务长株潭为发展路径，突出转方式、调结构，扩投资、强基础，抓改革、增活力，促增长、惠民生，确保全市经济社会又好又快发展。 　　今年我市经济社会发展的目标是：实现地区生产总值增长12%，工作目标13%，力争超过全省平均水平；第一、二、三产业增加值分别增长5%、13.8%、12%；规模以上工业增加值增长16%；全社会固定资产投资增长30%，工作目标40%；地方财政一般预算收入增长13%，社会消费品零售总额增长19%，城镇居民家庭人均可支配收入增长8%，农村居民家庭人均纯收入增长7%，人口自然增长率控制在7.3‰以内，城镇登记失业率控制在4.5%以内，亿元GDP生产安全事故死亡率控制在0.34以下，节能减排完成省定目标。 　　为此，今年要着力抓好以下七个方面的工作： 　　（一）加快推进新型工业化，着力转变发展方式 　　做大工业经济总量。继续开展“工业企业服务年”活动，加强重点骨干企业的运行监测和煤、电、油、运等生产要素的保障，稳定和提高产能。加强新竣工投产企业的跟踪服务，争取尽快达产见效。加强在建项目的推进力度，争取早建成、早投产。大力发展中小企业，重点支持市场前景好、成长性强的中小企业做大做强，力争新增规模工业企业100家，确保全年工业增加值增长14%以上。 　　促进产业结构升级。以提高增长质量和效益为重点，改造提升传统产业，抓好华菱涟钢产品结构调整及薄板深加工等项目，力争完成工业技改投资160亿元以上。抓紧实施十大产业集群规划，努力打造钢产品及深加工500亿产业集群，煤炭深加工100亿产业集群，有色冶炼及深加工、机械制造及铸造、建材、农机等4个50亿产业集群，煤机、农产品深加工、生物医药和工业陶瓷等4个10亿产业集群。 　　培育发展新兴产业。依托资源禀赋、产业基础和科技支撑，着力培育和发展先进制造、电子信息、新材料、新能源等新兴产业。按照建设“两型”社会要求，大力发展环保装备制造业、资源综合利用产业、洁净产品制造业和环境服务业。积极承接沿海和省内产业转移，发展壮大服饰、制鞋、皮革和食品、药品等终端消费品产业。 　　加快园区建设步伐。完善基础配套，加快标准厂房建设，确保全市园区固定资产投资增长40%以上。做好园区发展规划，强化功能定位，坚持特色兴园，加快产业集聚。进一步落实促进园区发展的政策措施，破解瓶颈，理顺体制，优化环境，加强考核，突出招商引资，主动对接央企和承接产业转移，推进项目建设，发展壮大园区经济，确保娄底经济开发区工业增加值增长30%以上，各县市区产业园工业增加值增长20%以上。 　　增强自主创新能力。继续开展“科技创新年”活动，实施人才强市战略，积极引进各类优秀人才。建材、煤机、农机、工业陶瓷各建立1个省级技术中心，加快组建锑品和工业陶瓷2个国检中心，组建钢铁、有色冶炼2大省级产业技术创新战略联盟。加大知识产权保护和专利申报力度，新增高新技术企业15家以上，高新技术产业增加值增长14%以上。推进“质量兴企、名牌强市”，支持湖南宜化等企业创建中国名牌产品。突出抓好国家高技术产业基地薄板深加工产业园建设，力争娄底经济开发区进入国家新型工业化示范基地行列，冷水江、涟源和双峰产业园进入省新型工业化示范基地行列。 　　大力推进节能减排。积极引进低碳技术，培育低碳产业，加快发展低碳经济。大力发展循环经济，着力抓好涟钢和五江集团2个省级循环经济试点企业，重点推进冷钢、湖南宜化、闪星锑业、海螺水泥等企业和县市区产业园循环经济试点工作。强化节能减排目标责任制，推进节能减排重点工程，加快固体废物无害化处置设施建设，年内启用市医疗废物处理处置中心，严格督促各类企业脱硫设施正常运行。加强管理节能、工程节能、结构节能和淘汰落后产能工作。有序开采矿产资源。加强生态建设和环境保护，进一步落实市人大常委会《关于加强环境保护的决定》，加强资水、涟水、孙水的环境保护和污染治理，圆满完成城市生活污水处理三年行动任务，以“四旁”（村旁、水旁、路旁、宅旁）绿化和退耕还林为重点，全面推进城乡绿化，努力建设秀美家园。 　　（二）切实推进项目建设，增强投资拉动作用 　　加强项目开发、争取和储备。继续开展“项目建设年”活动，突出对接长株潭的汽车制造、工程机械、电力机车、新能源、新材料、文化创意、休闲旅游和物流产业，精心策划一批战略性、基础性项目，开发重大产业项目50个以上，确保项目库更新率达到20%以上。重点争取安张衡铁路娄底段项目列入国家中长期铁路发展规划，湘中物流园、娄底民用支线机场、新化核电厂等重大项目列入国家和省“十二五”规划，全力做好塞海水库、娄底市水土保持工程、湘江流域重金属污染治理工程娄底项目区、全市中央下放煤矿棚户区搬迁避让工程、冷钢节能降耗改造、湖南省煤炭储备物流中心等项目的前期工作。 　　扎实抓好项目建设。确保完成全社会固定资产投资400亿元以上，力争430亿元，重点抓好105个重大建设项目。 　　交通基础设施项目完成投资90亿元以上，力争100亿元。抓好沪昆铁路客运专线娄底段、娄邵铁路扩能改造、安邵高速、娄新高速、新溆高速、娄益高速、娄衡高速、娄长高速、干线公路改造、冷水江火车东站、农村公路、天然气管道、成品油管道及油库加油站配套等项目建设。 　　工业项目完成投资180亿元以上，力争完成200亿元。抓好涟钢产品结构调整及薄板深加工、华菱安赛乐米塔尔汽车板电工钢、冷钢300万吨钢120万吨焦、金华车辆、涟钢机制公司轧辊生产线、农友机械、金峰机械、中兴液压、三星锻造、鸿帆铝业、中源新材料、红太阳电源新材料、华新水泥、涟源国产实业、湖南宜化60万吨尿素扩改、黑猫炭黑、湖南安石集团干熄焦余热发电技术改造、双峰凯迪生物质发电、双峰台资鞋业园成品鞋生产线、华润煤业、桑梓大跃煤矿、桥头河煤矿复采、新化工业陶瓷、双峰定园机械技改扩建、新化铁合金产业技改升级、汇源焦化煤气综合利用等项目建设。 　　城市基础设施项目完成投资25亿元以上。中心城区抓好S209线城区段改造、吉星路路网、甘桂路路网、沪昆高速公路连接线改扩建、鸿帆铝业天然气供气工程、珠山公园、氐星公园、市第二污水处理厂、市第二自来水厂输水干管复线等项目建设，推进各县市城区基础设施建设。 　　农林水利项目完成投资14亿元以上。抓好粮食增产工程、丰产油茶基地、中药材开发、黑猪和黑牛产业化、小Ⅰ型病险水库除险加固、退耕还林、城市防洪工程、白马和大梅山灌区续建配套与节水改造、饮用水源地保护、农村安全饮水、大中型水库库区移民基础设施等项目建设。 　　社会事业及其他项目完成投资10亿元以上。抓好市体育中心、市有线数字电视建设、市文化艺术中心，湖南人文科技学院、娄底技师学院（筹）、娄底卫校（升专）基础设施建设，新化、双峰、冷水江人民医院整体搬迁等项目建设。 　　完成房地产开发投资30亿元以上。 　　完成城镇以下固定资产投资60亿元以上。 　　努力拓宽项目筹融资渠道。争取中央和省资金25亿元以上，力争全市银行机构各项贷款新增100亿元以上，确保中小企业和“三农”贷款增长25%以上。争取年内娄星区农村信用合作联社改制组建农村商业银行，在新化筹建1家村镇银行，在冷水江、新化、双峰各建立1家小额贷款公司，引进股份制商业银行在我市设立分支机构，努力将我市打造成为全省首个“金融生态模范城市”。扩大省级上市企业后备库资源，重点推进娄底红太阳新材料和三泰轧辊两家公司上市步伐。加快推进投融资平台建设，进一步做大做强市城市建设投资集团有限公司，争取实现总融资18亿元以上。 　　科学编制“十二五”规划。认真总结“十一五”规划执行情况，深入分析“十二五”时期发展所处的新背景、面临的新情况，立足我市发展定位，对接融入和服务长株潭，集中民智，加强研究，在更高起点上科学谋划未来，加快我市建设“三基地一中心一枢纽”（新型能源原材料基地、特色装备与先进制造业基地、文化与生态旅游休闲基地、区域性商贸与物流中心、区域性交通枢纽）的进程。 　　（三）大力推进农业产业化，夯实“三农”发展基础 　　积极发展现代农业。加快推进农业规模化，加强基本农田建设与保护，遏制耕地抛荒，改造中低产田，确保粮食作物播种面积400万亩、总产165万吨以上。抓好粮食收购与储备工作，确保粮食安全。加快推进农业产业化经营，大力发展农民专业合作组织，积极推进土地依法有序流转，继续培植种养大户，抓好种养殖基地建设，积极发展农产品深加工产业，重点扶持一批实力强、来势好的龙头企业，确保农产品加工业产值增长20%以上。加快组建牧业、米业、特色水果、蔬菜、永丰酱业和林业等产业集团，争取年内创建1家国家级龙头企业。加快推进农业标准化工作，逐步建立农产品市场准入制度，加强动植物疫病防控及病虫害防治工作，强化农产品质量监管，确保农产品质量安全。提升气象、水文为“三农”服务的水平，增强防灾减灾能力。加快推进农业机械化，全市农业机械总动力达到260万千瓦以上。积极发展休闲农业、生态农业和观光农业，全面搞活农村经济。 　　促进农民持续增收。在促进农业产业化增加农民收入的同时，积极落实国家粮食直补、良种补贴、农机购机补贴等强农惠农政策，增加农民政策性收入。培训农民26万人次以上，实现农村劳动力转移就业110万人，增加农民工资性收入。落实各项扶持政策，鼓励支持返乡农民工自主创业和回乡创业，支持农民创业增收。加大扶贫和库区开发力度，加强库区移民扶持工作。 　　加快新农村建设。进一步完善“领导办点、部门帮扶、干部驻村”的工作机制，继续以78个省级新农村示范村建设为重点，市级重点打造6个新农村建设示范点（片），加大财政投入和涉农资金整合力度，加强村镇民居建设规划和管理，增强新农村建设的整体实效。落实市人大常委会《关于加快发展现代水利的决定》，全面完成17座纳入国家规划的小型水库除险加固等各项水利建设任务。加强农村道路、能源、水毁工程恢复和物流、通讯等设施建设，加快新化、涟源农村电网的改造。加快推进省级乡村清洁工程示范村建设，大力开展农村环境整治，着力改善村容村貌，逐步实现“布局优化、道路硬化、村庄绿化、环境美化”目标。 　　（四）努力推进新型城市化，提升区域经济承载能力 　　积极打造区域性中心城市。围绕建设“新型工业城市、交通枢纽城市、生态宜居城市、和谐文明城市”目标，切实提升城市承载力、带动力、辐射力和竞争力。完善城区规划，强化规划管理，严格规划执法。加快环线和干道改扩建，完善城市供水、供电、供气、客运、垃圾处理、污水处理等配套设施，力争三年内从根本上解决城市饮用水水质问题，基本实现城市污水、垃圾无害化处理全覆盖。启动国家园林城市、国家卫生城市和全国文明城市创建活动，加强城市管理，强化基层社区建设和基础工作，大力实施“净化、序化、绿化、亮化、美化”工程，提升城市品味，提高城市文明程度。 　　加快县市城区和小城镇扩容提质。推进冷水江城市东扩、涟源城市北扩东延战略实施，加快双峰城北新区、沿河风光带建设和新化环城西路、北塔综合区等重点工程建设。以重点镇和示范镇为突破口，加强小城镇规划、建设和管理，发挥小城镇联结城乡、辐射农村、扩大就业的功能。探索放宽城镇户籍限制，创造条件促进农业人口转移，加快城市化进程，充分发挥城市化在扩内需、调结构和促就业中的主动力作用。 　　（五）有效推进城乡消费，培育壮大第三产业 　　努力促进消费。继续推进“万村千乡市场工程”、“新网工程”、“双百市场工程”和农贸市场标准化改造，继续实施家电、汽车以旧换新和家电、汽车、摩托车下乡政策，扩大城乡消费。加快提升生活性服务业，积极引导和培育汽车、文化、保健、餐饮等消费热点，大力发展电子商务、物流配送、保险证券、信息咨询、会计法律等现代生产性服务业。大力发展楼宇经济、总部经济。加强住房公积金的管理和使用，加快普通商品住房建设，支持居民自住和改善型购房需求，进一步促进房地产业持续健康发展。 　　加快发展旅游业。巩固创建中国优秀旅游城市成果，改造旅游干线公路，进一步完善等级景区的服务配套体系，促进景区旅游、农业观光旅游、工业旅游协调发展，实现旅游接待人次突破700万，旅游总收入增长30%以上，着力将旅游业打造成为我市战略性支柱产业。积极推进曾国藩故里、大熊山、湄江、龙山等景区的统一开发和水府旅游示范区、仙女寨景区系列项目整体招商，实现景区建设大跨越。举办好“娄底（长沙）旅游文化宣传月”、“湖南大梅山文化旅游经济圈高峰论坛”等活动。加大旅游资源保护力度，落实《湖南省紫鹊界梯田梅山龙宫风景名胜区保护条例》，支持新化创建省级历史文化名城。 　　（六）深入推进改革开放，激发经济发展活力 　　抓好“两型”社会建设试点。加快编制《娄底市资源节约型和环境友好型社会建设综合配套改革试验实施方案》、《娄底市“两型”产业发展规划纲要》、《水府示范片区总体规划》和《水府示范片区起步区控制性详细规划》。抓紧产业项目的前期工作，跟踪推进“两型”产业项目建设。建立和完善资源产权制度，推进资源性产品价格改革，完善节能减排激励约束机制，创新资源开发管理机制，促进体制机制对接。积极开展“两型”示范创建活动。 　　积极扩大招商引资。组织好第三届中国中南建材家居博览会和首届娄商大会，积极参与上海世博会、湘台经贸洽谈会等重大招商经贸活动，力争一批机械与装备制造、新型能源原材料、生态休闲旅游、环保等重大产业项目落户我市。进一步创新服务方式，强化目标管理，完善奖惩机制，形成招商合力，注重招商实效，确保内联引资100亿元以上，实际利用外资1.5亿美元以上。扎实做好外事侨务工作，充分发挥港澳台同胞和海外侨胞的作用，积极引资引智。加强项目履约调度与跟踪，提高签约项目履约率。加快推进娄底铁路口岸建设，完善对外贸易基础条件，大力发展加工与服务贸易。 　　加快重点领域和关键环节改革。营造公平竞争的市场环境，鼓励、支持和引导非公有制经济持续健康发展。稳妥推进农村基础设施产权制度改革，完成集体林权制度改革，继续做好农村第二轮土地延包后续完善工作。推进医药卫生体制改革，重点做好娄星区、冷水江基本药物制度试点工作和公共医疗、基层医疗卫生事业单位绩效工资改革，探索建立覆盖城乡居民的基本医疗卫生服务体系。进一步完善国有资产监管体制，建立国有资产经营预算制度，强化企业改制后的社会管理，加快市属国有企业和省属下划企业的改制扫尾。全面完成市及县市区政府机构改革。积极推进财政“省直管县”改革，落实金融、投融资、价格等各项改革政策和措施。完善农村公路管养体制机制，巩固成品油价格和税费改革成果。 　　（七）持续推进民生改善，保持社会和谐稳定 　　积极促进就业再就业。引导和促进劳动密集型企业、中小企业、民营企业、各种服务业加快发展，鼓励和支持劳动者自主创业和自谋职业，鼓励高校毕业生到农村基层、中小企业就业和创业，新增城镇就业2.9万人，实现失业人员再就业1.6万人，继续保持城镇零就业家庭动态清零。切实维护劳动者特别是农民工的合法权益。 　　着力加强民生保障。落实城乡低保政策，逐步改善低收入群体的整体生活水平。做好失地农民的保障工作。推进娄星区新型农村养老保险试点工作，参保率达到80%以上，积极争取扩大试点覆盖面，着力保障农村老年居民基本生活。促进养老、医疗、工伤、生育、失业保险的扩面和基金征缴工作，新增城镇职工养老保险参保人数2.75万人，城镇居民医疗保险登记参保人数达到63万人以上。做好基本养老保险和基本医疗保险关系转移接续工作，保障流动就业人员的合法权益。继续推进保障性安居工程建设，新增廉租住房8000套、租赁补贴2500户，支持农村危房、棚户区改造。切实为民办好实事，让广大群众得到更多实惠。 　　大力发展社会事业。加快建设教育强市，重点加强基础教育，全面推行素质教育，加快发展职业教育，促进各类教育协调发展。深入实施义务教育经费保障体制改革，推动城乡教育资源共享，缩小城乡教育差距。加快基础教育设施建设，三年内解决城区读书难问题。逐步完善和发展农村学前教育，推进民办教育健康发展。支持湖南人文科技学院迎接教育部合格学校评估验收，支持娄底职业技术学院和潇湘职业学院发展，进一步加大娄底卫校升专和娄底技师学院筹建力度。推进教师绩效工资制度改革，加强教师队伍建设，提升办学质量和水平。加快社区卫生、农村卫生基本医疗服务体系建设，提高医疗保障水平，新型农村合作医疗参合率达到90%以上。加强疾病防控工作，提高突发性公共卫生事件的应急处置能力。做好红十字会工作。大力发展慈善事业和残疾人事业。全面推进文化强市战略，加强社会主义核心价值体系和精神文明建设，加快文化事业和文化产业发展，推进农家书屋、乡镇文化站和社区文化建设，加大网吧整治力度，继续做好广播电视村村通工程、农村公益电影放映和新闻出版工作，不断完善文化服务体系。深入开展全民健身活动，提高竞技体育水平，积极申办湖南省第12届运动会。做好人口和计划生育工作，健全完善管理机制，扎实开展优质服务，加强流动人口管理，提高出生人口素质，稳定低生育水平，降低出生人口性别比。强化食品和药品监管，确保公众饮食用药安全。继续开展全民国防教育和双拥共建活动，做好民兵预备役工作，密切军民关系。加强人民防空工作，发展老龄事业，重视革命老区工作，认真组织实施第六次人口普查，全面启动第二轮修志工作，做好民族、宗教、地震、对台、档案、保密等工作，重视和支持工会、共青团、妇联工作，大力发展妇女儿童事业。 　　切实维护社会稳定。创新信访维稳工作机制，开展“信访工作基础年”活动，强化信访责任，对重大信访问题实行跟踪督办、限期办结，着力解决信访突出问题，建好信访接待中心。强化安全发展理念，推进“平安乡镇”、“平安县市”创建活动，加快推进科技兴煤、科技保安步伐，加强瓦斯治理基础工作，提高煤炭开采机械化水平，实现煤矿安全生产形势持续好转。落实公共安全措施，提高群众安全防范意识。设立道路交通事故救助基金。继续深化道路交通、非煤矿山、烟花爆竹、危险化学品、特种设备、消防等重点行业领域安全专项整治，严防重特大事故发生。加强社会治安综合治理，夯实治安巡逻、电子监控、社区及农村警务等防控基础，做好人民调解工作，增强对违法犯罪的源头管控能力。集中打击暴力恐怖、黑恶团伙、黄赌毒和盗抢犯罪，进一步提高人民群众的安全感。

三、努力建设人民满意政府

　　面对新形势，迎接新挑战，完成新使命，必须按照“为民、务实、清廉、高效”的要求，着力提高驾驭市场经济、应对复杂局面的能力，着力提高科学、民主、依法行政水平。 　　严格依法行政。健全政府行政决策机制，使重大事项决策更加民主、科学、透明。实施政府规范性文件监督管理制度，规范行政执法行为，加强行政复议和行政应诉工作。认真落实市人大及其常委会各项决议决定，依法报告工作，自觉接受人大法律监督和工作监督，主动与政协加强协商沟通，接受政协的民主监督，广泛听取各民主党派、工商联、无党派人士和各人民团体的意见，努力提高人大议案、代表建议和政协建议、委员提案的办理质量和效率。加快电子政务建设，进一步推进政府信息公开，主动接受新闻舆论监督和人民群众的监督。 　　着力提高效能。以新一轮政府机构改革为契机，进一步理顺职责关系，健全协调配合机制，切实提高行政效能。进一步关注基层、重视基层、服务基层。继续深化行政审批制度改革，清理行政许可事项，规范行政审批行为，优化审批流程，缩短审批时限。加强机关规范化建设，精减会议和文件，减少和规范检查评比。开展政府绩效评估，落实行政问责，严格责任追究，加大监察、审计、督查力度，提高行政执行力，确保令行禁止、政令畅通。培育壮大财源税源，依法加强税收征管，努力增收节支，增强政府的配套支付和支出保障能力。 　　大力改进作风。坚持求真务实，执政为民，多干实事，少讲空话，真正把精力集中到抓发展、搞建设上来，集中到优服务、解难题上来，集中到为群众和基层谋利益、办实事上来。全面落实党风廉政建设责任制，扎实推进惩治和预防腐败体系建设，从制度上规范财政支付、土地和矿产资源开发、政府采购、国有资产等公共资源管理。严肃查处各类违法违纪案件，加大专项治理力度，坚决纠正损害群众利益的不正之风。大力提倡艰苦奋斗作风，坚决制止奢侈浪费，以务实廉洁的政府形象取信于民。 　　切实优化环境。严格落实《娄底市优化投资环境扩大招商引资的若干规定》等政策，建设好市政务服务中心，实行“一厅式”办公、“一条龙”服务和项目全程代理制度，逐步推行项目前期工作代理制，完善重大项目审批“绿色通道”，严格执行“一票制”刚性收费，严格落实首问责任制、岗位承诺制和限时办结制，努力打造高效、快捷、优质的政务环境。倡导亲商、安商、富商风尚，鼓励全民关注、支持、参与创业，加大行风评议力度，积极营造有利于投资创业的社会环境。严厉打击“三强”行为，集中整治企业周边环境，杜绝“索、拿、卡、要”现象，坚决净化项目建设和企业生产的外部环境。 　　各位代表，我市正处在科学发展、加速赶超的关键时期，新的形势催人奋进，新的征程任重道远。让我们在市委的正确领导下，深入贯彻落实科学发展观，团结带领全市人民，以更加宽广的视野谋划发展，以更加昂扬的斗志迎接挑战，以更加务实的作风勤勉工作，为圆满完成本次会议确定的目标任务，加速建设富裕文明和谐娄底而努力奋斗!
